# A Comparison of Two Fascial Plane Blocks for Abdominal Analgesia in Laparoscopic Cholecystectomy Surgery (M-TAPA vs. External Oblique Intercostal Plane Block): A Prospective Randomized Study

**DOI:** 10.3390/jcm14093050

**Published:** 2025-04-28

**Authors:** Bahadir Ciftci, Selcuk Alver, Birzat Emre Gölboyu, Mustafa Celalettin Haksal, Serkan Tulgar, Alessandro De Cassai, Haci Ahmet Alici

**Affiliations:** 1 Department of Anesthesiology and Reanimation, Istanbul Medipol University, Istanbul 34214, Turkey; bciftci@medipol.edu.tr (B.C.); selcukalver@yahoo.com (S.A.); 2 Department of Anatomy, Istanbul Medipol University, Istanbul 34815, Turkey; 3 Department of Anesthesiology, Katip Çelebi University, Izmir 35360, Turkey; birzatemre@windowslive.com; 4 Department of General Surgery, Istanbul Medipol University, Istanbul 34214, Turkey; 5 Department of Anesthesiology and Reanimation, Samsun Training and Research Hospital, Samsun University, Samsun 55090, Turkey; serkantulgar.md@gmail.com; 6 Department of Medicine—DIMED, University of Padua, 35121 Padua, Italy; 7 Institute of Anesthesia, University Hospital of Padua, 35128 Padua, Italy; 8 Department of Pain Medicine, Istanbul Medipol University, Istanbul 34083, Turkey; haalici68@gmail.com

**Keywords:** laparoscopic surgery, pain management, M-TAPA block, external oblique intercostal plane block

## Abstract

**Background:** Modified thoracoabdominal nerve block through a perichondrial approach (M-TAPA) and external oblique intercostal plane block (EOIB) provide abdominal analgesia by blocking thoracoabdominal nerves. Our aim was to compare the analgesic efficacy of M-TAPA vs. EOIB on the quality of recovery and pain scores in patients who underwent laparoscopic cholecystectomy surgery (LC). **Methods:** Patients with American Society of Anesthesiologists status I-II, aged between 18 and 65 years, and scheduled for elective LC under general anesthesia were enrolled in the study. The patients were randomized into two groups: Group M-TAPA (*n* = 30) and Group EOIB (*n* = 30). The blocks were performed with 40 mL 0.25% bupivacaine in total. The primary outcome of the study was the global quality of recovery score, and the secondary outcomes were the pain scores, rescue analgesic requirement, and adverse effects during the 24-h postoperative period. **Results:** The global quality of recovery scores at 24 h were similar in both groups. There was a reduction in the median static and dynamic numerical rating scale (NRS) in the first 2 h postoperatively for M-TAPA compared to the EOIB (*p* < 0.001). The need for rescue analgesia was significantly lower in the M-TAPA group compared to the EOIB group (*p* < 0.005). **Conclusions:** Opioid consumption was lower in the M-TAPA group, and the pain scores of the two groups were similar, with the exception of the first 2 h postoperatively. Both the M-TAPA block and EOIB are effective for analgesia following laparoscopic abdominal surgeries.

## 1. Introduction

Laparoscopically performed cholecystectomy (LC) is the cornerstone of modern surgical practice as it speeds up postoperative recovery [1]. Even if LC is a minimally invasive procedure, patients may experience severe pain in the postoperative period [2]. Such pain can occur due to inflammation as a result of surgical trauma, muscle and soft tissue trauma caused by port entry sites, insufflation of the abdominal cavity with carbon dioxide, and trauma to the liver caused by dissection of the gallbladder [3]. Inadequate analgesia in LC has implications for postoperative pain management and patient recovery, as it can lead to delayed mobilization and a longer hospitalization period [4]. PROSPECT guidelines recommend truncal blocks for post-LC analgesia [5,6]; however, various regional anesthesia techniques can be used to provide analgesia after laparoscopic procedures, though it is unclear which technique is superior. The suggested optimal target to provide analgesia using a regional anesthesia technique is represented by the T6–T10 thoracoabdominal nerves as they play a major role in pain after laparoscopic procedures [7,8]. Both external oblique intercostal plane block (EOIB) and modified thoracoabdominal nerve block through the perichondral approach (M-TAPA) represent valid alternatives to achieve this objective, but the superior technique remains a matter of debate [9,10,11]. M-TAPA is administered between the internal oblique and transversus abdominis muscles under the costal margin and provides wide dermatomal blockade in the anterior and lateral abdomen between the T4–T5 and T12-L1 levels [7,8,9,10,11,12]. The EOIB is administered between the external oblique muscle and rib/intercostal muscles at the level of the sixth to eight/ninth ribs and provides abdominal analgesia between the dermatome levels T7–T11/T12, predominantly in the lateral abdomen [8,13]. Both techniques are easy to perform, and there are no difficulties or major potential complications with either of them reported in the literature. A number of clinical studies have reported that these two blocks provide effective analgesia [14,15,16,17,18]. However, only one study has compared the analgesic efficacy of the two blocks [19].

The primary objective of this study was the global quality of recovery, assessed using the Turkish version of the Quality of Recovery-15 (QoR-15) questionnaire. Secondary objectives were to compare the pain ratings and opioid consumption in the first 24 h postoperatively.

## 2. Materials and Methods

### 2.1. Study Design and Patient Population

This study was a single-center, prospective, randomized trial conducted with the approval of the Istanbul Medipol University Ethics and Research Committee (decision no. 597, date 6 July 2022). Informed written consent was obtained from all participants. The trial was registered on ClinicalTrials.gov (NCT05502159) before the first patient was enrolled, and no modifications were performed after the registration. The study was conducted at Medipol Mega University Hospital between August 2022 and June 2023.

The study included patients aged 18–65 years with American Society of Anesthesiologists scores of I–II and scheduled to undergo LC under general anesthesia. The exclusion criteria were anticoagulant therapy, coagulopathy, allergies to local anesthetics, local infection in the area to be blocked, thoracic deformities, and an inability to comprehend or assess pain rating and recovery quality scoring systems.

### 2.2. Grouping, Randomization, and Blinding

Prior to undergoing surgery, the patients were divided into two groups (*n* = 30 in each groups): M-TAPA and EOIB. Utilizing the Research Randomizer computer application, randomization was carried out based on the generation of a randomization table and the assignment of a unique ID to each patient. Allocation concealment was granted by using the sealed opaque envelope technique. After informed consent and enrollment in the study, the researchers opened the envelope assigning the patient to the corresponding group.

During the study, the patients, surgical team, and nurse responsible for assessing postoperative outcomes were blinded to patient allocation.

### 2.3. Anesthesia Management and Surgical Technique

The standardized anesthesia protocol of our hospital was followed for the patients undergoing LC. As part of the multimodal analgesia regimen, all patients received 400 mg of intravenous (IV) ibuprofen and 100 mg of IV tramadol 20 min before the end of the surgery. In addition, 4 mg of IV ondansetron was administered to prevent postoperative nausea and vomiting.

All surgeries were performed by the same surgical team using the four-port technique, with the patients in a supine position. The positions of the main port sites were standardized as follows: infraxiphoidal (15–20 mm), intraumbilical (5 mm), junction of the anterior axillary line and right lateral side of the umbilicus (5 mm), and a symmetrical assistant port (5 mm). Intraabdominal pressure was set to 12–14 cmH_2_O as the standard and maintained.

### 2.4. Interfascial Plane Block Procedures

All blocks were performed under aseptic conditions with the patient in a supine position following the completion of the surgery and prior to extubation. A linear US transducer (11–12 MHz) and an 80 mm × 22-gauge block needle (Stimuplex Ultra 360; Braun, Germany) were used for the procedures.

#### 2.4.1. US-Guided M-TAPA Block

**Figure 1:**
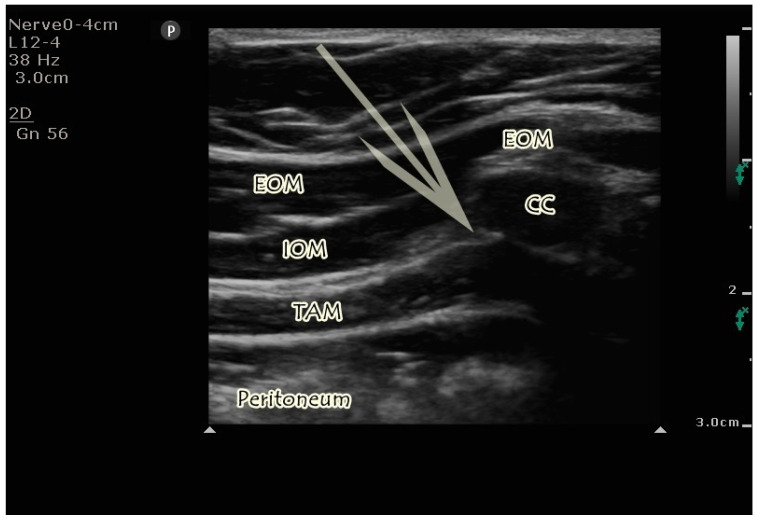
Sonographic visualization of M-TAPA. External oblique muscle, internal oblique muscle, transversus abdominis muscle, and tenth costal cartilage are seen. The arrow indicates the needle trajectory. The tip of the arrow is between the internal oblique muscle and transversus abdominis muscle. EOM; External oblique muscle, IOM; Internal oblique muscle, TAM; Transversus abdominis muscle, CC; Costal cartilage.

The transducer was positioned at the level of the ninth and tenth ribs on the costal cartilage in the sagittal plane, allowing visualization of the external oblique, internal oblique, and transversus abdominis muscles, as well as the tenth rib cartilage. The transducer angle was adjusted to enhance visualization of the underside of the costal cartilage. The block needle was inserted beneath the cartilage and directed into the plane between the transversus abdominis and internal oblique muscles (Figure 1). Isotonic solution (5 mL) was then injected to confirm needle placement, followed by the administration of 20 mL of 0.25% bupivacaine into the same plane just under the chondrium, with a total of 40 mL administered bilaterally.

#### 2.4.2. US-Guided EOIB

**Figure 2:**
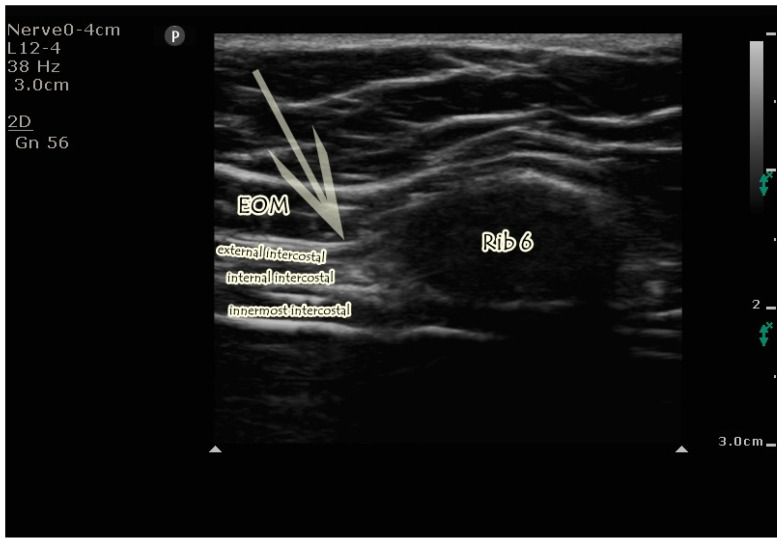
Sonographic visualization of EOIB. External oblique muscle, intercostal muscles, and the sixth rib are seen. The arrow indicates the needle trajectory. The tip of the arrow is between the external oblique muscle and intercostal muscles. EOM; External oblique muscle.

The transducer was placed on the tenth rib in the sagittal plane between the midclavicular and anterior axillary lines, and the ribs were counted upward to the sixth rib. By slightly rotating the transducer, a paramedian sagittal oblique view was achieved, with the cranial end positioned slightly medial and the caudal end slightly lateral. In this position, the external oblique muscle, rib, intercostal muscles, and pleura were visualized approximately 2 cm medial to the anterior axillary line. The needle was directed into the area between the external oblique and intercostal muscles at the sixth rib (Figure 2). Isotonic solution (5 mL) was then injected to verify needle placement, followed by 20 mL of 0.25% bupivacaine, with a total of 40 mL administered bilaterally. The block was performed by advancing the needle using hydrodissection from the sixth to the ninth ribs.

### 2.5. Postoperative Analgesia Management and Outcomes

Postoperatively, the patients were prescribed 400 mg of IV ibuprofen every 8 h. Pain levels were assessed using an 11-point numerical rating scale (NRS), where 0 indicated no pain and 10 represented the worst pain imaginable. NRS scores, both at rest and with movement, were recorded after 1, 2, 4, 8, 16, and 24 h. If a patient’s NRS score was 4 or higher, 0.5 mg/kg IV meperidine was planned as a rescue analgesic.

The primary outcome of the study was the global quality of recovery, assessed using the Turkish version of the Quality of Recovery-15 (QoR-15) questionnaire. Secondary outcomes included NRS scores, rescue analgesic/opioid consumption, and the occurrence of side effects, such as nausea, vomiting, and itching.

### 2.6. Sample Size

The sample size for this study was calculated using G*Power software (V.3.1.9). The primary objective was to compare QoR-15 scores between the groups. In a preliminary analysis involving eight patients per group, the postoperative QoR-15 scores were 144.5 in the M-TAPA block group and 142 in the EOIB group, with standard deviations (SDs) of 1.4 and 3.2, respectively. Based on an α error of 0.05 and a β error of 0.05, the required sample size per group was determined to be 27, providing a statistical power of 95%. To account for potential dropouts, a minimum of 30 patients per group was planned.

### 2.7. Statistical Analysis

The distribution of the variables (normal or skewed) in the study was assessed using the Shapiro–Wilk test. For normally distributed data, the results are presented as mean ± SD. Group comparisons were performed using independent samples *t*-tests. For nonparametric data, results are expressed as median and interquartile range, and group comparisons were performed using the Mann–Whitney *U* test. A *p*-value of less than 0.05 was considered statistically significant. All statistical analyses were performed using SPSS software version 25 (SPSS, Chicago, IL, USA).

## 3. Results

**Figure 3:**
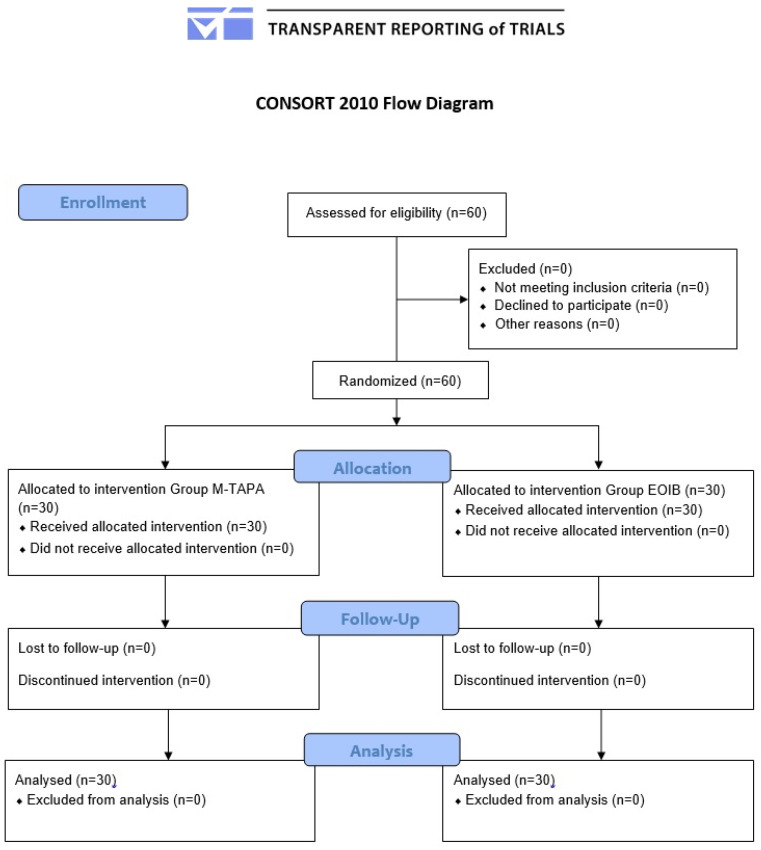
CONSORT flow diagram of the study.

Sixty patients were included, with 30 individuals in each group (Figure 3). Both groups were comparable in terms of demographic characteristics, as well as the length of the anesthesia and surgery (Table 1).

NRS scores at rest and during movement are presented in Table 2. The M-TAPA group had significantly lower NRS scores after the first and second postoperative hours (*p* = 0.019 and *p* = 0.015, respectively). However, no significant between-group differences were observed during the remaining postoperative 24-h period.

Table 3 displays rescue analgesia (meperidine) use. A significantly higher number of patients in the EOIB group required rescue analgesia (*n* = 12) compared to the M-TAPA group (*n* = 5) (*p* = 0.043), with opioid consumption notably higher in the EOIB group (*p* = 0.046).

No significant between-group differences were found regarding QoR-15 scores at the 24th postoperative hour (*p* = 0.062) (Table 4), and the incidences of nausea, vomiting, and pruritus were similar in both groups.

## 4. Discussion

The results indicated that NRS pain scores were lower in the M-TAPA group during the first two postoperative hours. Conversely, opioid consumption and the need for rescue analgesia were higher in the EOIB group within the first 24 h postoperatively. There was no significant difference in QoR-15 scores between the two groups.

Postoperative pain after LC surgery arises from three main sources: peritoneal inflation with carbon dioxide, port incision sites, and trauma to the liver caused by the cholecystectomy [2,16]. The ports are typically positioned in the infraxiphoid, intraumbilical, and right and left abdominal regions near the umbilicus. For effective sensory blockade in this area, it is crucial to block the medial and lateral cutaneous branches of the thoracoabdominal nerves at the T6–T11 levels [20]. The abdominal muscles—external oblique, internal oblique, and transversus abdominis—attach to the costal margin in the upper abdominal region. Specifically, the transversus abdominis attaches internally to the costal margin, the internal oblique attaches beneath it, and the external oblique extends over the costal margin from the tenth to the fifth to sixth ribs [7,8,20]. The thoracoabdominal nerves continue as intercostal nerves, branching off from the ventral rami of the spinal nerves in the anterolateral abdomen. These intercostal nerves divide into medial and lateral cutaneous branches, which lie between the transversus abdominis and internal oblique muscles, forming the (transversus abdominis plane) TAP plexus. The lateral branches pierce the external and internal oblique muscles in the lateral abdomen, and the medial branches extend anteriorly [18,19,20]. The M-TAPA block and EOIB work by targeting the course of these thoracoabdominal nerves in the anterolateral abdominal wall.

In the M-TAPA, the block is administered to the fascial plane between the transversus abdominis and internal oblique muscles, targeting the TAP plexus located just below the costal margin. This technique provides abdominal analgesia by blocking the medial and lateral branches of the thoracoabdominal nerves [7]. Although once believed to provide analgesia primarily to the medial abdomen, the M-TAPA block extends its effect to the lateral abdomen due to its application just beneath the costal margin [21,22,23]. The placement of the block in this region is crucial to its mechanism of action. When performing the M-TAPA block, directing the needle as cranially as possible is important to enhance multi-dermatomal abdominal analgesia via the endothoracic fascia, as there is no thick fascial barrier, like the linea semilunaris, to hinder the spread of the local anesthetic [16]. Ohgoshi et al. validated this mechanism in an anatomical study, where they observed local anesthetic spread within the space between the endothoracic fascia, diaphragm, and costodiaphragmatic recess in cadaver dissections [21]. Their findings were further supported by dermatome analysis in healthy volunteers, confirming sensory blockade between T8 and T12 in both the medial and lateral abdomen. Several clinical and cadaveric investigations have indicated that the M-TAPA offers efficacious abdominal analgesia in procedures such as LC and other abdominal surgeries [12,15,16,17,24,25,26,27,28,29,30]. Castillo-Davila et al., in clinical studies in Mexico, highlighted that incorporating the M-TAPA block into standard anesthesia protocols had economic benefits for middle-income countries, as it reduced opioid consumption and accelerated patient recovery [31].

The EOIB is based on the anatomical structure in which the external oblique muscle extends through the tenth costal margin and continues cranially to the sixth rib. This block is administered in the plane between the external oblique and intercostal muscles at the level of the sixth to ninth ribs, with the goal of allowing the local anesthetic to spread along this plane [8,9,13]. For the block to be effective, it is crucial to visualize the anesthetic spread in real-time using US. In patients where adequate spread is not achieved, a second needle may need to be inserted at a different rib level, utilizing the double-injection technique. The thoracoabdominal nerves form lateral cutaneous branches that innervate the lateral abdomen at the junction of the external oblique and serratus anterior muscles. These branches can be blocked by injecting local anesthetic between the external oblique and intercostal muscles at the sixth to seventh rib level. As the fascia of the external oblique muscle contributes to the formation of the anterior rectus sheath, local anesthetic can follow this fascial pathway to reach the rectus sheath, enabling blockade of the anterior cutaneous branches [18,20]. As a result, EOIB can provide dermatomal blockade at the T6–T10 levels, covering both the anterior and lateral abdomen [8,10]. Cadaveric studies have demonstrated local anesthetic spread to both the lateral and medial cutaneous branches [8,13]. In addition, studies have shown that EOIB provides effective analgesia in LC and other abdominal surgeries [14,18,19,32,33,34,35].

Although both M-TAPA and EOIB provide effective abdominal analgesia, there is ongoing debate in the literature on their respective effectiveness. Ohgoshi et al. reported in a dermatome analysis that the M-TAPA was effective in blocking the medial cutaneous branches and that the EOIB was effective in blocking the lateral branches at the T6–T11 levels [10]. However, in another study, the same authors found that M-TAPA provides only sensory blockade in both the medial and lateral abdomen [21]. Several dermatome analysis studies have similarly shown that the M-TAPA blocks both the medial and lateral cutaneous branches [22,24,25]. Elsharkawy et al. demonstrated in both a cadaver study and dermatome analysis that the EOIB was effective in blocking both lateral and medial cutaneous branches [8]. To the best of our knowledge, only one clinical study has compared the M-TAPA and EOIB [19]. In this clinical study on patients who underwent bariatric surgery, Turunc et al. found that the postoperative pain scores and opioid consumption of the patients in the M-TAPA and EOIB groups were comparable. In our study, although QoR-15 scores were similar in the two groups, pain scores were lower in the M-TAPA group during the first 2 h postoperatively, and opioid consumption was lower in the M-TAPA group overall. The EOIB may be easier to perform in obese patients, as it is more superficial and easier to visualize compared to the M-TAPA. Another point that should not be forgotten is that we did not use any agent to reverse the effect of opioid agents and anesthetic drugs used in our study. Opioid agents and analgesics used intraoperatively may also produce an analgesic effect postoperatively, but this effect is short-lived. In our study, the effect of the blocks was effective in the postoperative period of 24 h according to our results. In our study, intraabdominal pressure was standardized as 12–14 cmH_2_O. It should be kept in mind that different pressure levels will affect the pain level, and these pressure levels should be analyzed in different studies.

Our study has several limitations that warrant discussion. First, we used a 20 mL volume for the blocks; however, since fascial plane blocks are volume dependent, increasing the volume could have produced different results [36,37]. Second, the sample size was relatively small, and it could be underpowered for secondary outcomes analysis. Third, we did not performed dermatome analysis. An another limitation is the heterogeneity of gallbladder disease among patients. In a sample of 60 participants, variation in the severity and duration of disease is likely. Some patients may have experienced chronic biliary pain, which could modify postoperative pain sense due to changes in pain physiology. These differences in pain levels and responses may have affected analgesic requirements and pain scores, independent of the block technique. Finally, surgical factors such as intraoperative complications (e.g., bile extravasation), degree of adhesions, or technical difficulty—especially in overweight/obese patients—were not analyzed in this study. These factors could affect the level of postoperative pain and recovery and should be kept in mind in future research to provide a more comprehensive interpretation of analgesic effectiveness.

## 5. Conclusions

Although opioid consumption was lower in the M-TAPA group, the pain scores of the two groups were similar, with the exception of the first two hours postoperatively. Both the M-TAPA block and EOIB are effective for analgesia following abdominal surgeries, with the choice depending on the type of surgery and the anesthesiologist’s expertise.

## Figures and Tables

**Table 1:** Comparison of demographic data and durations of surgery and anesthesia between groups.

	Group EOIB (*n* = 30)	Group M-TAPA (*n* = 30)	*p*
Age	44 (37–48)	46 (37–52)	* 0.684
Gender (M/F)	13/17	16/14	**†** *1*
ASA (I/II)	14/16	10/20	**†** *0.430*
Height (cm)	170 (161–177)	169 (162–174)	* 0.796
Weight (kg)	81 (65–87)	78 (73–86)	* 0.589
Surgery length (min)	78 (65–99)	75 (65–91)	* 0.695
Anesthesia length (min)	94 (78–113)	90 (78–108)	* 0.673

Values are expressed median (percentiles 25–75) or number. * *p* value is obtained with Mann–Whitney U test. **†** *p* value is obtained with Pearson’s χ2 test (*n*). *p* values are italicized, and values that are written in bold represent statistical significance. ASA: American Society of Anesthesiologist, m: male, f: female, cm: centimeter, kg: kilogram, min: minutes.

**Table 2:** Comparison of static and dynamic NRS assessments between groups.

	Group EOIB (*n* = 30)	Group M-TAPA (*n* = 30)	*p*
At rest			
1st hour	1 (0–2)	0 (0–1)	***0.019***
2nd hour	1 (1–3)	1 (0–1)	***0.015***
4th hour	1 (1–2)	1 (0–2)	*0.066*
8th hour	1 (0–2)	1 (0–2)	*0.381*
16th hour	1 (0–1)	0 (0–1)	*0.177*
24th hour	0 (0–1)	0 (0–1)	*0.736*
During movement			
1st hour	2 (1–3)	1 (0–2)	***0.045***
2nd hour	3 (1–3)	1 (0–2)	***0.042***
4th hour	2 (1–2)	2 (1–2)	*0.085*
8th hour	2 (1–2)	1 (0–2)	*0.341*
16th hour	1 (0–2)	1 (0–1)	*0.211*
24th hour	0 (0–1)	0 (0–1)	*0.910*

Data are expressed as median (percentiles 25–75). *p* value is obtained with Mann–Whitney U test median (percentiles 25–75). *p* values are italicized, and values that are written in bold represent statistical significance. NRS: Numeric rating pain scale.

**Table 3:** The comparison of opioid consumptions (meperidine) and the need of rescue analgesia between groups.

	Group EOIB (*n* = 30)	Group M-TAPA (*n* = 30)	*p*
**Rescue analgesia** **(Y/*N*)**	12/18	5/25	***0.043***
**Rescue dose (mg)**	0 (0–60)	0 (0–0)	* 0.046

*p* value is obtained with Pearson’s χ2 test (n). Data are expressed as median. * *p* value is obtained with Mann–Whitney U test median (percentiles 25–75). *p* values are italicized, and values that are written in bold represent statistical significance. Y: yes (indicates the number of the participants that used rescue analgesia), N: No.

**Table 4:** Comparison of the incidence of side effects and QoR-15 scores between groups.

	Group EOIB (*n* = 30)	MTAPA Block (*n* = 30)	*p*
Nausea (Y/N)	10/20	5/25	*0.136*
Vomiting (Y/N)	4/26	3/27	*1*
Itching (Y/N)	6/24	2/28	*0.254*
Postoperative QoR-15	138 (128–140)	139 (137–140)	* 0.062

*p* value is obtained with Pearson’s χ2 test (*n*). Data are expressed as median.* *p* value is obtained with Mann–Whitney U test median (percentiles 25–75). *p* values are italicized, and values that are written in bold represent statistical significance. Y: yes, N: No. QoR: Quality of Recovery.

## Data Availability

The datasets generated and/or analyzed during the current study are not publicly available, but are available from the corresponding author on reasonable request.
